# Histamine receptor 1 is expressed in leukaemic cells and affects differentiation sensitivity

**DOI:** 10.1111/jcmm.15930

**Published:** 2020-10-20

**Authors:** Josep M. Cornet‐Masana, Antònia Banús‐Mulet, Laia Cuesta‐Casanovas, José M. Carbó, Francesca Guijarro, Miguel Ángel Torrente, Jordi Esteve, Ruth M. Risueño

**Affiliations:** ^1^ Josep Carreras Leukaemia Research Institute (IJC) Barcelona Spain; ^2^ Institut d’Investigació en Ciències de la Salut Germans Trias i Pujol (IGTP) Badalona Spain; ^3^ Faculty of Medicine University of Barcelona Barcelona Spain; ^4^ Faculty of Pharmacy University of Barcelona Barcelona Spain; ^5^ Faculty of Biosciences Autonomous University of Barcelona Barcelona Spain; ^6^ Institut d’Investigacions Biomèdiques August Pi i Sunyer (IDIBAPS) Barcelona Spain; ^7^ Department of Hematology Hospital Clínic Barcelona Spain

**Keywords:** differentiation, histamine, HRH1, leukaemia

## Abstract

Despite the success of immunotherapy in several haematological neoplasms, the effectiveness in acute myeloid leukaemia (AML) is still controversial, partially due to the lack of knowledge regarding immune‐related processes in this disease and similar neoplasias. In this study, we analysed the role and expression of histamine receptor 1 (HRH1) in haematological malignancies. Although the histamine receptor type 1 was widely expressed in healthy and malignant haematopoiesis, especially along the myeloid lineage, HRH1 lacked a relevant role in survival/proliferation and chemoresistance of AML cells, as analysed by HRH1 knockdown (KD) and pharmacological modulation. However, HRH1‐mediated signalling was critical for the activation of the differentiation process induced by several agents including all‐trans retinoic acid, establishing a role for HRH1 in myeloid differentiation. Pharmacological activation of Erk was able to partially restore differentiation capacity in HRH1 KD AML cells, suggesting that HRH1 signalling acts upstream MAPK‐Erk pathway. As an indirect consequence of our results, treatment‐related histamine release is not expected to confer a proliferative advantage in leukaemic cells.

Acute myeloid leukaemia (AML) is a haematological neoplasia characterized by the accumulation in bone marrow of transformed myeloid progenitors with enhanced proliferation and a block in differentiation. Indeed, AML cells are arrested at different stages of myeloid maturation.^1^ Among the plethora of known role‐players in myeloid differentiation, the histaminergic system has been recently described as a regulator of healthy and malignant haematopoiesis, mainly through the modulation by histamine of myeloid‐biased haematopoietic stem cell quiescence^2^ and the production of ROS in immature blasts.^3^, ^4^

Histamine is a pleiotropic biogenic amine acting through four different histamine receptors (HRH1 to HRH4) belonging to the G protein‐coupled receptor superfamily. Although most of the proposed effects of histamine on myeloid differentiation are mediated by HRH2,^2^, ^3^ the expression of HRH1 in AML cell lines has recently been reported.^5^ Nevertheless, no role for HRH1 in leukaemia has been identified.

To inquire on that open question, we first examined HRH1 expression in blood cell subsets from healthy donors, as well as in samples from patients with AML (Table S1) and the related myeloid malignancies chronic myelomonocytic leukaemia (CMML) (Table S2) and myelodysplastic syndromes (MDS) (Table S3). Regarding healthy cells, the expression differed greatly across subtypes; no expression was detected in primitive cord blood cells (CD45^+^ CD34^+^), T cells (CD45^+^ CD3^+^) and platelets (CD45^−^ CD41a^+^), while intermediate expression was observed for B cells (CD45^+^ CD19^+^) and granulocytes (CD45^+^ CD15^+^) (Figure 1A). Monocyte‐macrophage cells (CD45^+^ CD14^+^) showed a generalized HRH1 expression. Consistent with their molecular heterogeneity, HRH1 expression showed a wide distribution pattern in MDS and AML, ranging from 0% to 100%. Conversely, in CMML HRH1 expression was prevalent, reaching more than 50% in all 17 analysed samples (Figure 1A). These results, similar to healthy monocytes, are in agreement with the rather homogeneous nature of CMML cells, whose phenotype resembles differentiated monocytes.^6^ Similarly, in CMML, classical (CD14^+^ CD16^−^) and intermediate (CD14^+^ CD16^+^) monocytes are predominant,^6^ and they both consistently express HRH1, in contrast to non‐classical CD14^dim^CD16^+^ monocytes (Figure 1A). Given this HRH1 expression pattern in AML, we next interrogated the role of HRH1 signalling on cellular viability and clonogenic capacity both in primary AML samples and healthy donor cells. As we have previously described cytotoxic effects of several antihistamines based on their physicochemical properties and independently of HRH1,^5^ we selected fexofenadine and cetirizine, potent HRH1 antagonists devoid of these properties. In all healthy samples, agonism or antagonism of HRH1 produced no significant effects on the clonogenicity, while on neoplasic ones only histamine caused a slight reduction (Figure 1B,C). The exception was a slight bias towards granulocytic differentiation of umbilical cord blood (UCB)‐derived haematopoietic progenitor/stem cells (HPSCs) upon treatment with histamine (Figure 1D).

**FIGURE 1:**
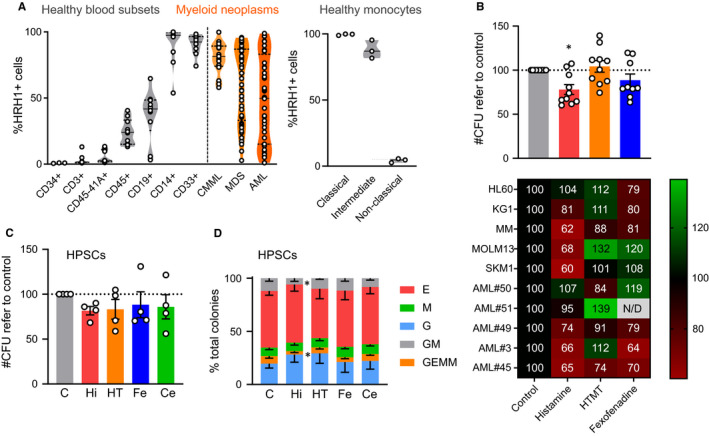
HRH1 expression in healthy and malignant haematopoiesis. A, HRH1 expression was analysed by flow cytometry in healthy blood cell subsets (n = 3‐10), CMML patient samples (n = 17), MDS patient samples (n = 45) and AML patient samples (n = 48). Each circle represents the mean value of each sample measured in triplicates. The right panel shows the expression of HRH1 in subsets of healthy‐donor monocytes (n = 3). Subsets were identified by marker phenotype (Classical, CD14^+^ CD16^−^; Intermediate, CD14^+^ CD16^+^, Non‐classical, CD14^dim^ CD16^+^). B, Clonogenicity assay for five AML primary samples and five AML cell lines treated with 500 µmol/L histamine (red bar), 10 µmol/L HTMT (orange bar, specific HRH1 agonist), 50 µmol/L fexofenadine (blue bar, specific HRH1 antagonist) or vehicle control (grey bar). Bars show mean ± SEM of the number of CFUS normalized to control. Each circle represents one sample (mean of a duplicate). Numbers of normalized CFUs are also represented in the lower heatmap‐coloured table. *P < .05 in a paired parametric *t* test using non‐normalized values. C, A clonogenic assay for HPSCs was performed with lineage‐depleted UCB cells (n = 4) treated with 500 µmol/L histamine (Hi, red), 10 µmol/L HTMT (HT, orange, specific HRH1 agonist), 50 µmol/L fexofenadine (Fe, blue, HRH1 antagonist), 50 µmol/L cetirizine (Ce, green, HRH1 antagonist) or vehicle control (C). Bars show mean ± SEM of the number of HPSC CFUs normalized to control. Each circle represents one sample (mean of a duplicate). D, Frequency of colony subtypes from (C). Error bars represent SEM. *P < .05 in a Two‐way ANOVA with Geisser‐Greenhouse correction

To further study the role of HRH1 expression by AML, we generated an HL60‐derived cell line stably expressing a CRISPR/Cas9‐mediated HRH1 down‐regulation system. The knockdown (KD) of HRH1 was assessed at a protein level both by flow cytometry and Western blot and was highly efficient (Figure 2A). KD of HRH1 resulted in a moderate increase in colony‐forming capacity (Figure 2B) and no significant changes in sensitivity to the commonly used chemotherapeutic cytarabine (Figure 2C) or histamine (Figure S1). Thus, HRH1 is probably not crucially involved in proliferation and chemosensitivity in HL‐60, although it may play a residual role in attenuating self‐renewal.

**FIGURE 2:**
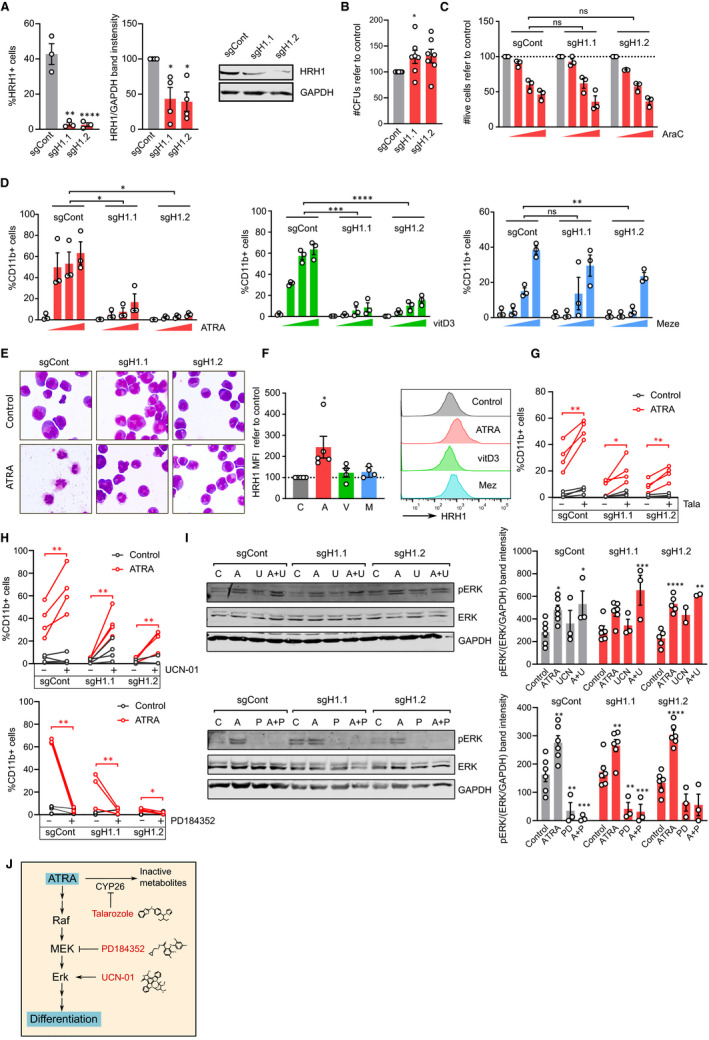
HRH1 affects sensitivity to ATRA‐induced differentiation. A, HL‐60 cells were transduced with pLentiCRISPR.v2 constructs, either scramble control (sgCont) or HRH1‐directed sgRNAs (sgH1.1, sgH1.2). Left panel: frequency of HRH1‐positive cells in the CRISPR‐transduced cell lines as assessed by flow cytometry. Each circle corresponds to an independent experiment (n = 3 in triplicates). **P < .01; ****P < .0001 in one‐way ANOVAs. Middle panel: HRH1 protein levels were quantified in four independent Western blots. Bars show mean ± SEM of the band intensity normalized to GAPDH and referred to control CRISPR. Each circle represents an independent experiment. *P < .05 in *t* tests. Right panel: representative Western blot membrane. B, Clonogenic capacity of CRISPR‐transduced HL‐60 cells. Bars show mean ± SEM of the number of CFUS normalized to control. Each circle represents an independent experiment (n = 7, mean of duplicates). *P < .05 in paired parametric *t* tests of non‐normalized data. C, CRISPR‐transduced HL‐60 cells were treated with vehicle control (grey) or with growing concentrations of cytarabine (3, 15 and 30 nmol/L AraC, red). Viability was assessed by flow cytometry after 48 h. Bars show mean ± SEM of live cells refer to control. Each circle represents an independent experiment (n = 3 in triplicates). Significance was assessed by a 2‐way ANOVA. D, CRISPR‐transduced HL‐60 cells were treated for 72 h with vehicle control (grey) or growing concentrations of ATRA (red; 0.5 1and 2 µmol/L), vitamin D3 (green; 1, 10 and 100 nmol/L) or mezerein (blue; 0.1, 1 and 5 nmol/L). After that time, differentiation was assessed by the frequency of CD11b^+^ cells as detected by flow cytometry. Bars show mean ± SEM. Each circle corresponds to an independent experiment (n = 3 in triplicates). *P < .05; **P < .01; ***P < .001; ****P < .0001 in 2‐way ANOVAs. E, Representative images of CRISPR‐transduced HL‐60 cells treated for 72 h with control vehicle or 2 µmol/L ATRA and stained with May‐Grünwald‐Giemsa solution. F, HL‐60 cells were treated for 72 h with vehicle control (C, grey) 0.5 µmol/L ATRA (A, red), 100 nmol/L vitamin D3 (V, vitD3, green) or 1 nmol/L mezerein (M, Mez, blue) and HRH1 levels were analysed by flow cytometry. Left panel shows the normalized mean fluorescence intensity of HRH1 staining. Five independent experiments were performed. Each circle corresponds to the mean of triplicates from a single experiment. *P < .05 in a paired *t* test. Right panel shows a representative flow cytometry plot. G, CRISPR‐transduced HL‐60 cells were treated for 72 h with vehicle control (grey) or 1 µmol/L ATRA (red) in the presence or absence of 2.5 µmol/L talarozole (Tala). After that time, differentiation was assessed by the frequency of CD11b^+^ cells as detected by flow cytometry. Each pair of circles connected by a line correspond to an independent experiment (n = 4 in triplicates). * *P* < .05; ** *P* < .01 in paired *t* tests. H, CRISPR‐transduced HL‐60 cells were treated for 72 h with vehicle control (grey) or ATRA (red; upper panel 0.5 µmol/L, lower panel 2 µmol/L) in the presence or absence of 80 nmol/L UCN‐01 (upper panel) or 2 µmol/L PD184352 (lower panel). After that time, differentiation was assessed by the frequency of CD11b^+^ cells as detected by flow cytometry. Each pair of circles connected by a line corresponds to an independent experiment (upper panel n = 4; lower panel n = 3 in triplicates). * *P* < .05; **P < .01 in 2‐way ANOVAs. I, CRISPR‐transduced cells were treated for 24 h with vehicle control (C), 2 µmol/L ATRA (A), 100 nmol/L UCN‐01 (U, UCN), 2 µmol/L PD184352 (P, PD) or the indicated combinations of them (A + U, A + P) and their proteins were extracted and subjected to Western blots analysing phospho‐ERK, total ERK and GAPDH. Representative membranes are shown (left panel). Bands were quantified and protein levels (pErk/(Erk/GAPDH)) are represented as means 2‐6 biological replicates ± SEM. All values are normalized to the mean of all experiments and circles represent values from each replicate. *P < .05; **P < .01; ***P < .001; ****P < .0001 in two‐way ANOVAs with Dunnett's multiple comparison test. J, Schematic representation of the pharmacological modulation used in the experiments

As one of the main features of AML is the maturation blockade and some effects of histamine have been observed on myeloid differentiation,^7^ we next interrogated the effects of HRH1 signalling on sensitivity to differentiation‐inducing agents. We treated control and HRH1‐KD AML cells with three different differentiation‐inducing agents modulating diverse pathways; the all‐trans retinoic acid (ATRA), vitamin D3 and the phorbol ester mezerein. Contrary to cytarabine sensitivity, HRH1 KD resulted in a drastic decrease in ATRA‐ and vitamin D3‐induced differentiation and a milder effect on response to mezerein, as assessed by surface expression levels of the CD11b (Figure 2D) and CD14 (Figure S2) myeloid‐associated differentiation markers. This was also observed for viability and clonogenicity (Figures S2). At a morphological level, HRH1 KD did not elicit in any major alteration in the basal state, while the decrease in ATRA response could also be confirmed (Figure 2E). As HRH1 surface level recapitulated the maturation status of blood cells (Figure 1A) and its signalling modulated the response to differentiation stimuli (Figure 2D), we further investigated the receptor trafficking during the differentiation process. ATRA exposure resulted in a more than 2‐fold increase in HRH1 expression, while no significant effects in vitamin D3‐ and mezerein‐treated cells could be observed (Figure 2F). HRH2 was slightly up‐regulated in the presence of ATRA in HRH1‐KD cells (Figure S3A), whereas HRH3 and HRH4 are not expressed on these cells (Figure S3B). These observations strengthened the relationship between HRH1 signalling and myeloid differentiation and suggested that it may rely on specific pathways, given the fact that only ATRA exposure could elicit an increase in HRH1.

To seek for mechanistic explanations for the role of HRH1 in AML differentiation, we probed publicly available AML expression data in the R2 Genomics (https://hgserver1.amc.nl), performing a GSEA analysis on the top genes correlated with HRH1 in 4 different AML datasets. The KEGG retinol metabolism gene set recurrently appeared on top of the analyses (Figure S4). Given our previous results regarding ATRA response and previous studies highlighting the pro‐differentiation effects on AML of inhibiting CYP26, a key player in retinol metabolism,^8^ we next combined ATRA and the CYP26 inhibitor talarozole in control and HRH1‐KD HL60 cells. By inhibiting CYP26, we prevent the metabolization of ATRA to inactive hydroxylated forms and force an increase in ATRA intracellular concentration.^9^ As shown in Figure 2G, talarozole could partly rescue the decrease in ATRA response observed in HRH1‐KD cells, suggesting that effects on retinoid metabolism were, at least partially, involved in the lower response to ATRA.

As MAPK pathway activation is considered a key event in ATRA‐induced differentiation of HL‐60,^10^ we also analysed its relationship with HRH1‐KD. Pharmacological Erk activation by UCN‐01 was able to restore the HRH1‐KD differentiation capacity, while the MEK inhibitor PD184352 abrogated their residual differentiation at higher doses of ATRA (Figure 2H). These effects had their correlates with the levels of Erk phosphorylation (Figure 2I,J).

In our study, we described an increase in HRH1 surface levels along healthy myeloid differentiation. Accordingly, the more differentiated monocyte‐like CMML cells showed high levels of HRH1, similarly to classical and intermediate monocytes. On the contrary, AML and MDS, neoplasms with highly misregulated myeloid differentiation showed lower and heterogeneous HRH1 expression as compared with healthy myeloid cells. Rather than playing a lead role in myeloid malignancies, HRH1 may thus reflect the maturation state of abnormally differentiated cells. Then, the lower levels of HRH1 observed in AML and MDS could be related to the low levels of HRH1 seen in HSCs and non‐myeloid blood cells, as previously reported (Figure 1A).^2^ On the other hand, pharmacological modulation or CRISPR‐mediated KD of HRH1 did not induce significant effects on chemosensitivity or basal differentiation state, while histamine induced only a moderate reduction in clonogenic capacity, thus precluding the therapeutic potential of HRH1 targeting in myeloid malignancies and further supporting a minor role of HRH1, in line with previous results.^5^ Despite the lack of effects of HRH1 modulation in leukaemogenesis, our results establish a role for HRH1 in myeloid differentiation. Surprisingly and contrary to the otherwise inert effect of HRH1 depletion, HRH1‐KD HL60 cells were refractory to differentiation, in sharp contrast with parental HL60. In addition, this was proven to be a general differentiation refractoriness, as the phenomenon was observed upon treatment with 3 different stimuli acting through separate signalling pathways. Mechanistically, we have hypothesized that the metabolism of ATRA could be involved, as a decrease in CYP26 activity is able to partially overcome the block in ATRA‐induced differentiation. CYP26 has been previously involved in HSC self‐renewal through bone marrow microenvironment.^11^ Also, HRH1‐KD effects are probably acting upstream of MAPK signalling, because its pharmacological modulation is able to restore the lost differentiation capacity. Complementarily, HRH1 was highly up‐regulated upon ATRA treatment, strengthening the link between HRH1 and ATRA sensitivity. This link, then, works both ways: HRH1 is increased during differentiation, and at the same time is crucial for differentiation to take place.

Lately, chimeric antigen receptor (CAR) T cells have been proposed as promising strategies to eradicate AML. One of the main side effects associated with those therapies is the cytotoxicity resulting from an excessive immune activation known as cytokine release syndrome, managed, among others, with antihistamines.^12^, ^13^, ^14^ Even if our results demonstrate HRH1 expression in blasts from many AML patients, they also show that neither histamine nor antihistamines promote AML cell growth, discarding any relevance in the CAR‐T cell context.

Taken together, the results obtained in this study suggest an involvement of HRH1 in healthy and malignant myeloid differentiation, that will need to be mechanistically elucidated in future studies.

## CONFLICT OF INTEREST

RMR is a shareholder of Leukos Biotech.

## AUTHOR CONTRIBUTIONS

**Josep Maria Cornet‐Masana:** Conceptualization (equal); Data curation (lead); Formal analysis (lead); Investigation (lead); Methodology (lead); Validation (equal); Writing‐original draft (equal); Writing‐review & editing (equal). **Antònia Banús‐Mulet:** Data curation (supporting); Formal analysis (supporting); Investigation (supporting); Methodology (supporting); Validation (equal); Writing‐review & editing (supporting). **Laia Cuesta‐Casanovas:** Data curation (supporting); Formal analysis (supporting); Investigation (supporting); Methodology (supporting); Writing‐review & editing (supporting). **Jose Maria Carbó:** Data curation (supporting); Formal analysis (supporting); Investigation (supporting); Methodology (supporting); Validation (equal); Writing‐review & editing (supporting). **Francesca Guijarro:** Conceptualization (supporting); Methodology (supporting); Resources (equal); Writing‐review & editing (supporting). **Miguel Ángel Torrente:** Conceptualization (supporting); Formal analysis (supporting); Methodology (supporting); Resources (equal); Writing‐review & editing (supporting). **Jordi Esteve:** Conceptualization (supporting); Methodology (supporting); Resources (lead); Supervision (equal); Writing‐review & editing (equal). **Ruth M. Risueño:** Conceptualization (lead); Data curation (equal); Formal analysis (equal); Funding acquisition (lead); Investigation (supporting); Methodology (equal); Project administration (lead); Resources (equal); Supervision (lead); Visualization (lead); Writing‐original draft (equal); Writing‐review & editing (lead).

## Supporting information

Supplementary Material

## Data Availability

All data and materials are available upon reasonable request to Ruth M. Risueño (risueno@carrerasresearch.org).
