# Supplementary material for: Histamine receptor 1 is expressed in leukaemic cells and affects differentiation sensitivity
Source: J Cell Mol Med. 2020 Oct 20;24(22):13536–41. doi: 10.1111/jcmm.15930 (PMC7701509; doi:10.1111/jcmm.15930)
Supplement: Supplementary file 1 — Supplementary Material [file JCMM-24-13536-s001.docx]

**SUPPORTING INFORMATION**

**SUPPLEMENTARY MATERIALS AND METHODS**

**Cell culture**

Cell lines HL-60 (ACC-3), KG-1 (ACC-14), THP-1 (ACC-16), Kasumi-1 (ACC-220) and MonoMac-1 (ACC-252) were obtained from DSMZ. 293T cell line (CRL-3216^TM^) was obtained from ATCC. SKM-1 and Molm-13 were kindly provided by Dr. Marcus Buschbeck (Josep Carreras Leukaemia Research Institute). Cell lines were cultured in RPMI (AML cell lines) or DMEM (293T) supplemented with heat-inactivated fetal bovine serum (FBS, Lonza), 2 mM L-Glutamine (Lonza) and/or non-essential amino acids (Lonza) according to manufacturers’ recommendations. Primary AML blasts were cultured in IMDM (Biowest) supplemented with 3% heat-inactivated FBS, 2 mM L-Glutamine, 20% BIT 9500 Serum Substitute (StemCell Technologies), 5 ng/ml IL3 (Peprotech), 1 mM sodium pyruvate (Gibco) and 5 x 10^-5^ M β-mercaptoethanol (Sigma-Aldrich) and 0.1 mM non-essential amino acids. Lineage depleted UCB cells were cultured in IMDM supplemented with 0,1% Bovine Serum Albumin (BSA) (Sigma), 100 ng/mL SCF (Peprotech), 100 ng/mL FLT3L (Peprotech), 20 ng/mL TPO (Peprotech), 5 x 10^-5^ M β-mercaptoethanol, 1 mM sodium pyruvate and 0.1 mM non-essential amino acids.

**Primary samples**

Primary samples were obtained from patients diagnosed at Hospital Clínic of Barcelona (Spain) and Hospital Germans Trias i Pujol (Badalona, Spain). AML, MDS and CMML diagnosis and classification was based on standard WHO criteria (Arber et al., 2016). Main patients’ characteristics are summarized in supplementary Table 1 (AML), 2 (CMML) and 3 (MDS). Samples were obtained from bone marrow, peripheral blood or leukapheresis and mononuclear cells (MNCs) and were isolated by Ficoll density gradient centrifugation (GE). All patients provided written informed consent in accordance with the Declaration of Helsinki, and the study was approved by the Ethics Committee of Hospital Clínic of Barcelona and Hospital Germans Trias i Pujol. Mature blood MNCs were isolated from healthy-donor buffy coats provided by Banc de Sang i Teixits (Barcelona, Spain). Umbilical cord blood was provided by Banc de Sang i Teixits (Barcelona, Spain). MNCs were obtained after Ficoll density gradient centrifugation and were depleted for lineage marker-positive cells using magnetic separation with human Lineage Cell Depletion kit (Miltenyi Biotec) following manufacturer’s recommendations.

**Drugs**

All drugs were resuspended in H_2_O (Thermo Fischer Scientific) or DMSO (Sigma-Aldrich) according to manufacturer’s specifications and were stored at -80ºC at 10 mM. histamine dihydrochloride, HTMT dimaleate and cetirizine dihydrochloride were obtained from Tocris. Cytarabine, mezerein, 1α,25-Dihydroxyvitamin D3 (vitamin D3), all-trans retinoic acid (ATRA), PD184352, UCN-01 and R115866 (talarozole) were obtained from Sigma-Aldrich. Fexofenadine hydrochloride was obtained from Santa Cruz Biotechnology.

**HRH1 expression**

Primary samples were stained with HRH1 rabbit polyclonal antibody ABIN1530769 (Antibodies Online) and the secondary antibody P-phycoerythrin goat anti-rabbit IgG (H+L) (Life technologies). In healthy samples, HRH1 expression analysis was performed in each cell subset, as assessed by the indicated markers. Analyses in malignant samples were performed inside the CD45^dim^ SSC^int^ gate, in order to identify blasts.

**Clonogenicity assay**

1 x 10^3^ cells of AML cell lines or 50 x 10^3^ primary AML cells or lineage-depleted UCB cells were treated at the indicated concentration for 18 h, and cultured in 1 mL of MethoCult H4034 Optimum (StemCell Technologies). Colonies were screened based on morphology and cellularity at day 7 (cell lines) or day 14 (AML primary cells and lineage-depleted UCB cells).

**CRISPR-mediated HRH1 knockdown**

For HRH1 knockdown, 2 sgRNA guides cloned in pLentiCRISPR.v2 vector were obtained from Genscript (guides ATCTGAGTCCGTTCGAGAGA and GTACCGGCATACAGCACCAGC). Lentivirus were generated in HEK293T cells, and HL-60 cells were transduced by centrifugation and hexadimethrine bromide treatment. Transduced cells were selected by puromycin resistance. Knockdown was validated by flow cytometry and western blot. Transduction with empty pLentiCRISPR.v2 was used as a control.

**Western blot**

Cells treated in the indicated conditions were harvested, lysed in RIPA lysis buffer, subjected to SDS-PAGE and transferred to a 0.2 µm nitrocellulose membrane. HRH1 was detected using PA5-27817 rabbit anti-human HRH1 antibody (Thermo Fisher Scientific). Phospho-Erk was detected using mouse anti-human phospho-p44/42 MAPK (Erk1/2; Thr202/Tyr204) clone E10 antibody (#9106, Cell Signaling Technologies). Total Erk was detected using rabbit anti-human p44/42 MAPK (Erk1/2) antibody (#9102, Cell Signaling Technologies). GAPDH, used as a loading control, was detected using mouse anti-human GAPDH antibody (clone 6C5, Thermo Fisher Scientific).

#

As secondary antibodies, IRDye® 800CW Donkey Anti-Rabbit IgG (H+L) and IRDye® 680RD Donkey Anti-Mouse IgG (H+L) (LI-COR) were used. Images were acquired in a LI-COR Odyssey CLx Imager and quantified using Fiji software.

**Cytotoxicity assay**

2 x 10^5^ cells per mL were cultured in 96-well plates in their corresponding medium and all drugs were added at the indicated concentrations. Cell viability was measured by 7-AAD (eBioscience) exclusion by flow cytometry, and cell count was obtained by volume.

**Differentiation assay**

Cells were treated at the indicated drug concentrations for 72 h. PE-conjugated anti-human CD11b (clone ICRF44, BD Pharmingen) was used as a myeloid differentiation marker. For specific monocytic differentiation, FITC-conjugated anti-human CD14 (clone M5E2, BD Pharmingen) was used. Samples were measured by flow cytometry.

### **May–Grünwald–Giemsa staining**

Cells were treated in the indicated conditions. After treatment, cells were harvested, washed with PBS + 0.5 mM EDTA and attached to slides using Cytospin 4 (Thermo Scientific, Wealtham, MA, USA). The slides were then stained for 20 minutes with May-Grünwald’s eosin-methylene blue (Merck) followed by 5-minute staining with Giemsa’s azure-eosin-methylene blue 20% (Merck). Images were acquired in an Olympus BX53 microscope, at a x60 magnification.

**Semi-Quantitative Real Time PCR**

Total RNA of cells treated in the indicated conditions was isolated using the *Total RNA Purification Kit* (Norgen Biotek) following the manufacturer’s reccomendations. 1 µg RNA was subsequently reversely transcribed to cDNA using the *qScript cDNA Synthesis Kit* (Quanta Biosciences). RealTime PCRs were performed using the *PowerUp SYBRGreen MasterMix* (Applied Biosystems) and the following primer pairs:

HRH2

Fw: 5’ CCATCCTGCATGACACCAAA 3’

Rv: 5’TCTCCGCTTCCCAGGTTTT 3’

HRH3

Fw: 5’ TCGTGCTCATCAGCTACGAC 3’

Rv: 5’ AAGCCGTGATGAGGAAGTAC 3’

HRH4

Fw: 5’ GGCTCACTACTGACTATCTG 3’

Rv: 5’ CCTTCATCCTTCCAAGACTC 3’

GAPDH

Fw: 5’ gtggacctgacctgccgtct 3’

Rv: 5’ ggaggagtgggtgtcgctgt 3’

GUSB

Fw: 5’ AAACGATTGCAGGGTTTCAC 3’

Rv: 5’ CAGTCATGAAATCGGCAAAA 3’

The reaction and detection was done in a *Step One Plus Real-Time PCR System* (Applied Biosystems), and results were analyzed using the machine software. GAPDH was used as the reference housekeeping gene. Results are presented as Fold Changes (2^-ΔΔCt^) normalized to GAPDH and untreated controls. Only Ct<35 were considered.

**Flow cytometry**

All flow cytometry data were acquired with a FACSCanto II cytometer (Becton Dickinson) and analyzed using FlowJo software (TriStar).

# Statistical Analysis

# Statistical significance was determined using GraphPad Prism® 6.01 (GraphPad software) by the statistical tests specified in figure legends. Error bars correspond to SEM. All experiments were done at least 3 times in biological triplicates, unless otherwise specified in the figure legend.

# Gene Set Enrichment Analysis

All the analyses of publicly available expression data were performed in the R2: Genomics Analysis and Visualization Platform (<http://r2.amc-nl>). Microarray expression data were obtained from GSE37642 (Bohlander, 422 samples), GSE14468 (Verhaak, 524 samples), GSE13159 (MILE study, 516 AML samples) and GSE6891 (Delwel, 461 samples). First, the correlation of HRH1 (205580_s_at and 205579_at probes) with every other single gene in the dataset was calculated in the platform (each dataset and probe were analysed independently). Next, a Gene Set Analysis was performed based on KEGG gene sets, and sets with a p-value ≤ 0.01 were considered.

**SUPPLEMENTARY FIGURE AND FIGURE LEGENDS**

**Supplementary Figure 1. HRH1 KD doesn’t affect histamine sensitivity.** CRISPR-transduced HL-60 cells were treated for 24h with 500 µM histamine (light blue), 2 µM ATRA (red) or a combination of both (orange) and a clonogenic assay was performed. Statistical significance was tested by 2-way ANOVAs.

**Supplementary Figure 2. HRH1-KD cells are less sensitive to differentiation agents.** CRISPR-transduced HL-60 cells were treated for 72h with vehicle control (grey) or increasing concentrations of ATRA (red; 0.5 µM, 1 µM and 2 µM) and vitamin D3 (green; 1 nM, 10 nM and 100 nM). After that time, viability was assessed by volume count of 7AAD-negative cells (upper panels), and monocytic differentiation was assessed by the frequency of CD14 cells as detected by flow cytometry (lower panels). Bars show mean±SEM. Each circle corresponds to an independent experiment (n=3 in triplicates). *p<0.05; **p<0.01; in 2-way ANOVAs.

**Supplementary Figure 3. HRH1 KD has a minor impact on other histamine receptors.** (**A**). CRISPR-transduced HL-60 cells were treated for 24h with 2 µM ATRA (red) or vehicle control (grey). RNA was subsequently isolated and reversely transcribed, and HRH2 mRNA levels were analyzed by a semi-quantitative RealTime PCR, using GAPDH as a reference. *p<0.05 in t-tests. (**B**). cDNA from five AML cell lines was used as a template for semi-quantitative RealTime PCRs using primers for HRH3, HRH4 and GUSB detection. Positive controls are cDNA from ReNcell neural progenitor cell line (HRH3) and healthy-donor blood MNCs (HRH4).

**Supplementary Figure 4.** **Retinol metabolism is correlated with HRH1 in AML datasets.** A GSEA analysis was performed for genes correlated with HRH1 in AML publicly available expression data using the Genomics Analysis and Visualization Platform. Bars represent the -log10 (p-value) of KEGG terms with a p-value ≤ 0.01. Results are represented independently for each dataset, and retinol metabolism is marked in red.

**SUPPLEMENTARY TABLES AND TABLE LEGENDS**

**Supplementary Table 1**

| **ID** | **Sample** | **Sex** | **Age** | **WHO subtype** | **WBC** | **% PBB** | **% BMB** | **Karyotype** | **Molecular Alterations** | **Risk** | **ChR** |
| --- | --- | --- | --- | --- | --- | --- | --- | --- | --- | --- | --- |
| #1 | BM | M | 49 | AML with myelodysplasia-related changes | 76.4 | 42 | 26 | 46-47,XY,del(5)(q22q34),del(6)(q22q25),del(7)(q22q23), -8,-9,add(11)(q23),+i(11)(q11),  -16,+mar1,+mar2,+mar3[cp8] | None detected | Adverse | Yes |
| #2 | LA | M | 48 | AML NOS | 131.6 | 63 | 81 | 46,XY[17] | None detected | Interm | No |
| #3 | LA | F | 44 | AML with mutated *NPM1* | 159 | 76 | 80 | N/A | NPM1mut | Favorable | No |
| #4 | PB | F | 57 | AML with maturation | 3.97 | 4 | 41 | 46,XX[20] | None detected | Interm | No |
| #5 | LA | F | 67 | MPAL, T/Myeloid, NOS | 92.7 | 53 | 30 | 46,XX[21] | None detected | Adverse | Yes |
| #6 | PB | F | 80 | AML with mutated *NPM1* | 172.6 | 53 | 95 | 46,XX[20] | FLT3-ITD, NPM1mut | Interm | No |
| #7 | BM | M | 41 | AML with t(3;3)(q21;q26) | 15.49 | 57 | 73 | 46,XY,t(3;3)(q21;q26)[1]/45,X,-Y,t(3;3)(q21;q26)[19] | None detected | Adverse | Yes |
| #8 | BM | F | 45 | AML with minimal differentiation | 2.72 | 0 | 20 | 46,XX[20] | None detected | Interm | No |
| #9 | BM | M | 64 | AML with mutated *NPM1* | 1.7 | 56 | 78 | 46, XY[20] | NPM1mut | Favorable | No |
| #10 | PB | F | 90 | AML with myelodysplasia-related changes | 338 | 92 | 77 | 42,XX,del(5)(q22q34),del(7)(q22q32),add(8)(q24),-15,-16,add(17)(p13),-18,-19,-20,-21,-22,-22,+r(?),+mar[cp18] | TP53mut | Adverse | No |
| #11 | LA | F | 63 | AML with mutated *NPM1* | 384.2 | 100 | 94 | N/A | NPMmut  FLT3-ITD, DNMT3Amut, IDH2mut | Interm | No |
| #12 | PB | M | 43 | AML NOS | 13.06 | 17 | 47 | 46,XY[21] | DNMT3A mut, TET2mut, CEBPAmut mono | Interm | No |
| #13 | PB | M | 63 | AML with myelodysplasia-related changes | 2.69 | 43 | 30 | N/A | TP53mut | Adverse | Yes |
| #14 | BM | M | 27 | N/A | 3.1 | N/A | N/A | 46,XY[20] | None detected | Interm | No |
| #15 | BM | M | 57 | N/A | 1.4 | 43 | 33 | 46,XY [5] | None detected | Interm | No |
| #16 | BM | M | 73 | N/A | 0.5 | 0 | 27.3 | N/A | FLT3-ITD | Adverse | No |
| #17 | LA | M | 47 | AML with mutated *NPM1* | 118 | 23 | 48 | Normal | NPMmut  DNMT3Amut, IDH2mut, TP53mut | Adverse | No |
| #18 | PB | M | 55 | AML with minimal differentiation | 2.22 | 46 | 55 | 46,XY[20] | MLL-PTD | Interm | Yes |
| #19 | PB | F | 26 | AML with t(6;9)(p23;q34) | 1.31 | 5 | 22 | 46,XX,t(6;9)(p23;q34)[10]/46,XX[10] | None detected | Adverse | N/A |
| #20 | PB | F | 54 | AML with minimal differentiation | 67.29 | 47 | 83 | 48,XX,+add(13)(q34),+add(13)(q34)[8]/46,XX[5] | None detected | Interm | Yes |
| #21 | PB | F | 24 | AML with t(6;11)(q27;q23) | 10.54 | 60 | 80 | 46,XX,t(11;6)(q23;q27)[18] | None detected | Adverse | No |
| #22 | BM | M | 48 | AML with myelodysplasia-related changes | 76.5 | 42 | 26 | 46-47,XY,del(5)(q22q34),del(6)(q22q25),del(7)(q22q23),-8,-9,add(11)(q23),,+i(11)(q11),-16,+mar1,+mar2,+mar3[cp20] | None detected | Adverse | Yes |
| #23 | BM | F | 81 | AML with myelodysplasia-related changes | 3.84 | 2 | 22 | 46,XX[20] | None detected | Interm | N/A |
| #24 | BM | F | 55 | AML with mutated *NPM1* | 3.03 | 0 | 3 | 46,XX[20] | NPM1mut | Favorable | Yes |
| #25 | BM | M | 70 | N/A | 3.00 | 0 | 39 | 47,XY,+8[12] / 48,XY,+8,+8[7] / 46,XY[2] | None detected | Interm | No |
| #26 | BM | M | 45 | N/A | 4.10 | N/A | N/A | 46, XY[20] | FLT3-ITD | Adverse | No |
| #27 | BM | M | 71 | AML with mutated *NPM1* | 4.30 | N/A | N/A | N/A | NPM1 mut | Favorable | No |
| #28 | BM | M | 27 | AML with myelodysplasia-related changes | 2.01 | 3 | 34 | 46,XY[37] | FLT3 -ITD | Interm | No |
| #29 | PB | M | 69 | AML with myelodysplasia-related changes | 5.83 | 23 | 3 | 43,XY,-4,add(4)(q?35),del(5)(q13q33),-7,der(12)t(12;?14)(p?12;q?12),-14,-15,del(20)(q11q13),-21,+mar1,+mar2[22] | None detected | Adverse | N/A |
| #30 | BM | F | 82 | AML with mutated *NPM1* | 119.3 | 79 | 83 | 46,XX,der(12)t(1;12)(q21;q24.3)[4] / 46,XX[12] | NPM1 mut | Favorable | Yes |
| #31 | BM | M | 78 | N/A | 10.2 | N/A | N/A | N/A | None detected | Interm | No |
| #32 | BM | M | 73 | N/A | 0.5 | 0 | 27.3 | N/A | None detected | N/A | No |
| #33 | BM | M | 45 | N/A | 4.50 | N/A | 0.33 | 46, XY[20] | None detected | Adverse | No |
| #34 | BM | M | 70 | N/A | 2.8 | 0 | 5 | 46,XY[20] | None detected | Interm | No |
| #35 | BM | M | 78 | N/A | 11.3 | N/A | N/A | N/A | None detected | Interm | No |
| #36 | BM | M | 27 | N/A | 2.8 | N/A | N/A | 46,XY[20] | None detected | Interm | No |
| #37 | N/A | N/A | N/A | N/A | N/A | N/A | N/A | N/A | N/A | N/A | N/A |
| #38 | N/A | N/A | N/A | N/A | N/A | N/A | N/A | N/A | N/A | N/A | N/A |
| #39 | N/A | N/A | N/A | N/A | N/A | N/A | N/A | N/A | N/A | N/A | N/A |
| #40 | PB | F | 64 | AML without maturation | 2.21 | 48 | 73 | 46,XX[30] | MLL-PTD, IDH2mut, DNMT3Amut | Adverse | No |
| #41 | PB | F | 69 | AML with mutated *RUNX1* | 5.3 | 68 | 66 | 47,XX,+8[20] | NRASmut, RUNX1mut, CEBPAmut mono | Adverse | No |
| #42 | PB | F | 74 | AML with mutated *NPM1* | 3.41 | 40 | 78 | 46,XX,+8[25] | FLT3-TKD, ASXL1mut | Favorable | N/A |
| #43 | PB | F | 77 | AML with mutated *NPM1* | 40.70 | 95 | 85 | N/A | FLT3-ITD, TET2mut | Interm | N/A |
| #44 | PB | M | 62 | AML with mutated *RUNX1* | 51.16 | 49 | 81 | 6,XY,del(7) [19]/46,XY[1] | None detected | Adverse | No |
| #45 | BM | M | 49 | AML with myelodysplasia-related changes | 76.4 | 42 | 26 | 46-47,XY,del(5)(q22q34),del(6)(q22q25),del(7)(q22q23),-8,-9,add(11)(q23),+i(11)(q11),-16,+mar1,+mar2,+mar3[cp8] | None detected | Adverse | Yes |
| #46 | LA | M | 47 | AML with mutated *NPM1* | 118 | 23 | 48 | 46, XY[20] | DNMT3Amut, IDH2mut,  TP53mut | Adverse | No |
| #47 | LA | M | 40 | AML with mutated *NPM1* | 109 | 96 | 93 | N/A | IDH1mut, NPM1mut, FLT3-ITD | Adverse | N/A |
| #48 | BM | F | N/A | N/A | N/A | N/A | N/A | N/A | N/A | N/A | N/A |
| #49 | LA | M | 65 | AML with mutated *NPM1* | 107 | 62 | 80 | 46, XY[20] | NPM1mut, FLT3-ITD | Favorable | No |
| #50 | PB | M | 74 | AML NOS with monocytic differentiation | 102 | 93 | 65 | 45,X,-Y[4]/46,X,-Y,+8[2]/46,XY[19] | NRASmut, ASXL1mut | Interm | No |
| #51 | PB | F | 45 | AML NOS with monocytic differentiation | 20 | 25 | 91 | 46, XX[20] | MLL-PTD, FLT3-ITD, RUNX1mut, TET2mut, DNMT3Amut | Interm | No |

**Supplementary Table 1. AML patients’ information.** Abbreviations: WBC, white blood cell (x10^9^/L); PBB, peripheral blood blasts; BMB, bone marrow blasts; ChR, chemorefractory; BM, bone marrow; PB, peripheral blood; LA, leukapheresis; mut, mutant; N/A, not available; NOS, Not otherwise specified; MPAL, mixed phenotype acute leukemia; Interm: intermediate; Interm, intermediate.

| **ID** | **Sex** | **Age** | **WHO subtype** | **Karyotype** | **IPSS-R** | **%BMB** |
| --- | --- | --- | --- | --- | --- | --- |
| #1 | M | N/A | CMML | N/A | N/A | N/A |
| #2 | F | N/A | CMML -1 | 47,XX,+21[13] | N/A | N/A |
| #3 | M | 72 | CMML-0 | 46,XY,del(5)(q31q33)[6] | N/A | 0 |
| #4 | F | 59 | CMML-0 | 46,XX[20] | N/A | 2 |
| #5 | M | 71 | CMML-0 | 46,XX[20] | N/A | 0 |
| #6 | M | 76 | CMML-0 | 46,XY[20] | Very Low | 2 |
| #7 | M | 66 | CMML-0 | 46,XY,t(4;16)(q21;q24)[3]/47,sl,+8[17] | N/A | 0 |
| #8 | M | 66 | CMML-0 | 46,XY[20] | N/A | 4 |
| #9 | M | 76 | CMML-1 | 46,XY[20] | N/A | 1 |
| #10 | M | 52 | CMML-1 | 46,XY[20] | N/A | 4 |
| #11 | M | 60 | CMML-1 | N/A | N/A | 0 |
| #12 | F | 70 | CMML-1 | 46,XX[20] | N/A | 1 |
| #13 | M | 70 | CMML-1 | 46,XY[20] | N/A | N/A |
| #14 | M | 80 | CMML-1 | 46,XY[20] | N/A | N/A |
| #15 | M | 56 | CMML-1 | 47,XY,+8[14]/46,XY[3] | N/A | N/A |
| #161 | M | 69 | CMML-1 | 46,XY[20] | N/A | N/A |
| #17 | M | 79 | CMML-2 | 46,XX[20] | N/A | 13 |

**Supplementary Table 2**

**Supplementary Table 2. CMML patients’ information.** Abbreviations: BMB, bone marrow blasts; IPSS-R, revised international prognostic scoring system; N/A, not available.

**Supplementary Table 3**

| **ID** | **Sex** | **Age** | **WHO subtype** | **Karyotype** | **IPSS-R** | **%BMB** |
| --- | --- | --- | --- | --- | --- | --- |
| #1 | F | 73 | MDS del(5q) | 46,XX,del(5)(q22q33)[15]/46,XX[5] | Low | 2 |
| #2 | F | 71 | MDS del(5q) | 46 XX,-11,+mar [20] | Intermediate | 3 |
| #3 | F | 29 | MDS del(5q) | 46,XX,del(5)(q12q32)[11]/46,XX[9] | N/A | N/A |
| #4 | F | 59 | MDS del(5q) | 46,XX,del(5)(q13q33)[4]/46,XX[15] | Low | 2 |
| #5 | M | 71 | MDS del(5q) | 46,XY,del(5)(q14q34)[8]/46,XY[22] | Low | 7 |
| #6 | M | 78 | MDS del(5q) | 46,XY,del(5)(q13q33)[12]/46,XY[8] | Very Low | 2 |
| #7 | F | 80 | MDS del(5q) | 46,XX,del(5)(q13)[11]/46,XX[9] | Low | 4 |
| #8 | F | 94 | MDS del(5q) | 46,XX,del(5)(q13q33)[9]/46,XX[24] | Very Low | 1 |
| #9 | F | 63 | MDS del(5q) | del(5q) add(7) | Low | 1 |
| #10 | F | 64 | MDS del(5q) | 46,XX del(5)(q15q33)[20] | Low | 2 |
| #11 | F | 59 | MDS del(5q) | 46,XX,del(5)(q22q31-32)[5]/46,XX[15] | Low | 2 |
| #12 | M | 91 | MDS-EB1 | 45,X,-Y[14]/46,XY[6] | Intermediate | 7 |
| #13 | F | 67 | MDS-EB1 | 26/08/2015: 46,XX,del(5)(q11q31)[15] | Low | 8 |
| #14 | M | 79 | MDS-EB1 | 46,XY[21] | Intermediate | 5 |
| #15 | F | 53 | MDS-EB1 | 46,XX,del(5)(q14q33)[7]/46,XX [13] | Intermediate | 8 |
| #16 | F | 83 | MDS-EB1 | del(7)(q22q31) | Very High | 5 |
| #17 | M | 83 | MDS-EB-1 | 47,XY,+8[3]/46,XY[28] | High | 7 |
| #18 | M | 74 | MDS-EB2 | 45,X,-Y,del(1)(p13p32),der(11)t(Y;11)(q11;q13)[8]/46,XY[12] | Very High | 12 |
| #19 | M | 77 | MDS-EB2 | Complex | Very High | >5% |
| #20 | M | 67 | MDS-EB2 | 43,XY,-5,der(13;14)(q10;q10),add(15)(p10),add(16)(q24),-17,add(17)(p13),-18,+mar[cp14] | Very High | 15 |
| #21 | F | 53 | MDS-EB2 | N/A | High | 12 |
| #22 | M | 54 | MDS-EB2 | 46,XY,t(16;17)(q24;q22) | Very High | 12 |
| #23 | F | 46 | MDS-MLD | 46,XX[20] | Low | 2 |
| #24 | F | 70 | MDS-MLD | 46,XX[20] | Intermediate | 4 |
| #25 | M | 51 | MDS-MLD | N/A | N/A | 2 |
| #26 | F | 81 | MDS-MLD | 46,XX[10] | Low | 0 |
| #27 | F | 86 | MDS-MLD | 47,XX,+8[10]/48,idem,+mar [2]/46,XX[7] | High | 3 |
| #28 | M | 67 | MDS-MLD | 46,XY[20] | Low | 4 |
| #29 | F | 60 | MDS-MLD | 46,XX[20] | Very Low | 1 |
| #30 | M | 83 | MDS-MLD | 46,XY[20] | Very Low | 1 |
| #31 | M | 75 | MDS-MLD | 47,XY,+21[5]/46,XY[15 | Low | 3 |
| #32 | F | 78 | MDS-MLD | 46,XX,del(5)(q22q35),del(11)(q13.1q23.3)[17]/46,XX[3] | Low | 4 |
| #33 | M | 56 | MDS-MLD | 46,XY[20] | Low | 2 |
| #34 | M | 80 | MDS-MLD | 46,XY[20] | Low | 0 |
| #35 | F | 73 | MDS-MLD | 46,XX,add(9)(p24),16qh+c[7]/46,XX,16qh+c[7] | Low | <2 |
| #36 | F | 68 | MDS-MLD | 46,XX, del(5)(q13q33),del(11)(q13q23) | Low | 1 |
| #37 | M | 73 | MDS-MLD | 46,XY[2] | Low | 2 |
| #38 | F | 70 | MDS-MLD | 46,XX[20] | Very Low | 1 |
| #39 | F | 75 | MDS-MLD | 46,XX,-5,-14,+mar1,+mar2[16]/45,XX,del(5)(q13q33),-6[2]/46,XX[8] | High | 1 |
| #40 | F | 30 | MDS-MLD | 46,XX[20] | Very Low | 2 |
| #41 | M | 65 | MDS-MLD-RS | 46,XY[20] | Very Low | 2 |
| #42 | M | 65 | MDS-MLD-RS | 46,XY[20] | Very Low | 2 |
| #43 | F | 69 | MDS-MLD-RS | del(7) | N/A | N/A |
| #44 | F | 78 | MDS-RS | 46,XX[20] | Very Low | 0 |
| #45 | M | 58 | MDS-SLD | 46,XY,inv(2)(p23q13),del(5)(q13q32) | Low | 1 |

**Supplementary Table 3. MDS patients’ information** Abbreviations: BMB, bone marrow blasts; IPSS-R, revised international prognostic scoring system; N/A, not available; MDS-EB, MDS with excess blasts; MDS-MLD, MDS with multilineage dysplasia; RS, ring sideroblasts; MDS-SLD, MDS with single line dysplasia.

**References**

Arber, D. A., Orazi, A., Hasserjian, R., Thiele, J., Borowitz, M. J., Le Beau, M. M., … Vardiman, J. W. (2016). The 2016 revision to the World Health Organization classification of myeloid neoplasms and acute leukemia. *Blood*, *127*(20), 2391–2405. https://doi.org/10.1182/blood-2016-03-643544
